# Paving the Way From the Lab to the Field: Using Synthetic Microbial Consortia to Produce High-Quality Crops

**DOI:** 10.3389/fpls.2018.01467

**Published:** 2018-10-05

**Authors:** Zhaoyu Kong, Miranda Hart, Hongguang Liu

**Affiliations:** ^1^Key Laboratory of Poyang Lake Environment and Resource Utilization, School of Life Science, Ministry of Education, Nanchang University, Nanchang, China; ^2^Department of Biology, University of British Columbia Okanagan, Kelowna, BC, Canada; ^3^Jiangxi Provincial Key Laboratory of Soil Erosion and Prevision, Jiangxi Institute of Soil and Water Conservation, Nanchang, China

**Keywords:** plant growth-promoting bacteria, arbuscular mycorrhizal fungi, biostimulants, metabolite, nutrients, ecological impacts, microbial network analysis, core microorganisms

## Introduction

Crop quality is of increasing concern with the expanded demands from consumers. Recently, increasing attention has been paid on the crops rich in mineral nutrients, antioxidants, or other metabolites, as they represent high quality and reduce the risk of chronic diseases (Li and Eunice, 2015; Timmusk et al., 2017). Such high-quality crops are more profitable for farmers compared with conventional crops. However, the methods to improve crop quality are limited to breeding, fertilizing regimes, and farming practices.

Soil microbiome significantly contributes to the fitness improvement of plants, in facing abiotic/biotic stress and nutritional deficiency (Bakker et al., 2018; Oyserman et al., 2018). Crop quality can be potentially modified by the soil microbiome. However, conventional farming practices, e.g., tillage, over use of chemical fertilizers, pesticide and fungicide, and monoculture, disturb the soil microbiome. The overuse of agro-chemicals is especially detrimental to agricultural ecosystems, threatening soil quality and human health (Hartman et al., 2018). Crop quality may subsequently decline with the degradation of the soil microbiome. Hence, it is urgent to search for alternative methods to produce high-quality crops in an efficient, safe and environment-friendly manner.

Beneficial soil microbes, such as plant growth promoting bacteria (PGPB), actinomycetes, and arbuscular mycorrhizal fungi (AMF), can interact with plants and induce the accumulation of plants' metabolites which benefit people's health (Gianinazzi et al., 2010; Glick, 2012). Hence, using microbial biostimulants may be useful for producing high-quality crops sustainably (Bhardwaj et al., 2014). However, it is not easy to replace the agro-chemicals by biostimulants to produce nutritional crop safely. Natural rhizosphere communities are complex and diverse, comprising an entire food web (Bender et al., 2016). Commercially available biostimulants, however, are generally limited to one, or few, microbial taxa. While these products have yet to be thoroughly tested, it is unlikely that they will sufficiently compensate for reduced microbial diversity in farmlands due to human activity (Hart et al., 2018).

Borrowing from the ideas of synthetic biology, synthetic microbial consortia (SMC) could potentially replace and/or reshape the structure and function of plant microbiome. It is possible to construct SMC consisting of a microbial guild (rather than limited microbial taxa in the existing biofertilizers) with multiple functions to promote crop growth and quality (Wallenstein, 2017). In this regard, using SMC could potentially solve the drawbacks of traditional biofertilizers (Qin et al., 2016), such as ineffectiveness in competing with indigenous microbes, incompatibility with host plants and inadaptation to the local conditions (Hart et al., 2018). Here, we summarize how SMC may be used to produce high-quality crops, focusing on: (1) constructing the desired SMC; (2) assessing the efficacy of SMC; (3) assessing ecological impacts of SMC.

## How to construct the desired SMC?

Previously, developing SMC was largely based on combining specific microbial genotypes with desirable traits (Whipps, 2001; Dodd and Ruiz-Lozano, 2012; Thijs et al., 2014). Currently, the typical SMC often include PGPB and AMF, targeting to enhance the metabolites contents (e.g., essential oil, zein, glucosinolate, sugar, ascorbic and folic acid, volatile compounds, vitamin, and anthocyanin) and nutrients (N, Ca, P, Mg, K, Na, Fe, Mn, Cu, Zn, and B), which represent higher nutraceutical values in crops (Hart and Forsythe, 2012; Berta et al., 2014; Cosme et al., 2014; Bona et al., 2015, 2017; Hart et al., 2015; Weisany et al., 2015; Battini et al., 2016; Torres et al., 2016; Avio et al., 2017). However, previous studies dealing with SMC have reported a range of plant responses and contradictory results (Lucas García et al., 2004; Estévez et al., 2009; Rosier et al., 2016), suggesting that different microbes may not have additive effects. Importantly, the compatibility within microbes and with new environment is an essential consideration for constructing SMC. Given the variability among microbes and soil heterogeneity, this is no small task. Clearly, the way forward must capitalize on existing co-adapted SMC.

Firstly, the origin of microbes is critical to construct SMC. Indigenous microbes were reported to be more efficient in augmenting plant stress tolerance (Estrada et al., 2013; Armada et al., 2014; Ortiz et al., 2015). The environmental adaptation of autochthonous microbes might underlie their ability to improve plant fitness. Thus it is expected that the soil microbiome from high-quality crops is an ideal origin for SMC to confer the same plants better growth and quality. Further, the rhizosphere is a hotspot for selecting members for SMC, due to their intensive interactions with plants. Moreover, the endophytes beneficial to plants can also be used to devise SMC (Huang et al., 2018), since they are more likely to persist in environments (Kong and Glick, 2017).

**Figure 1:**
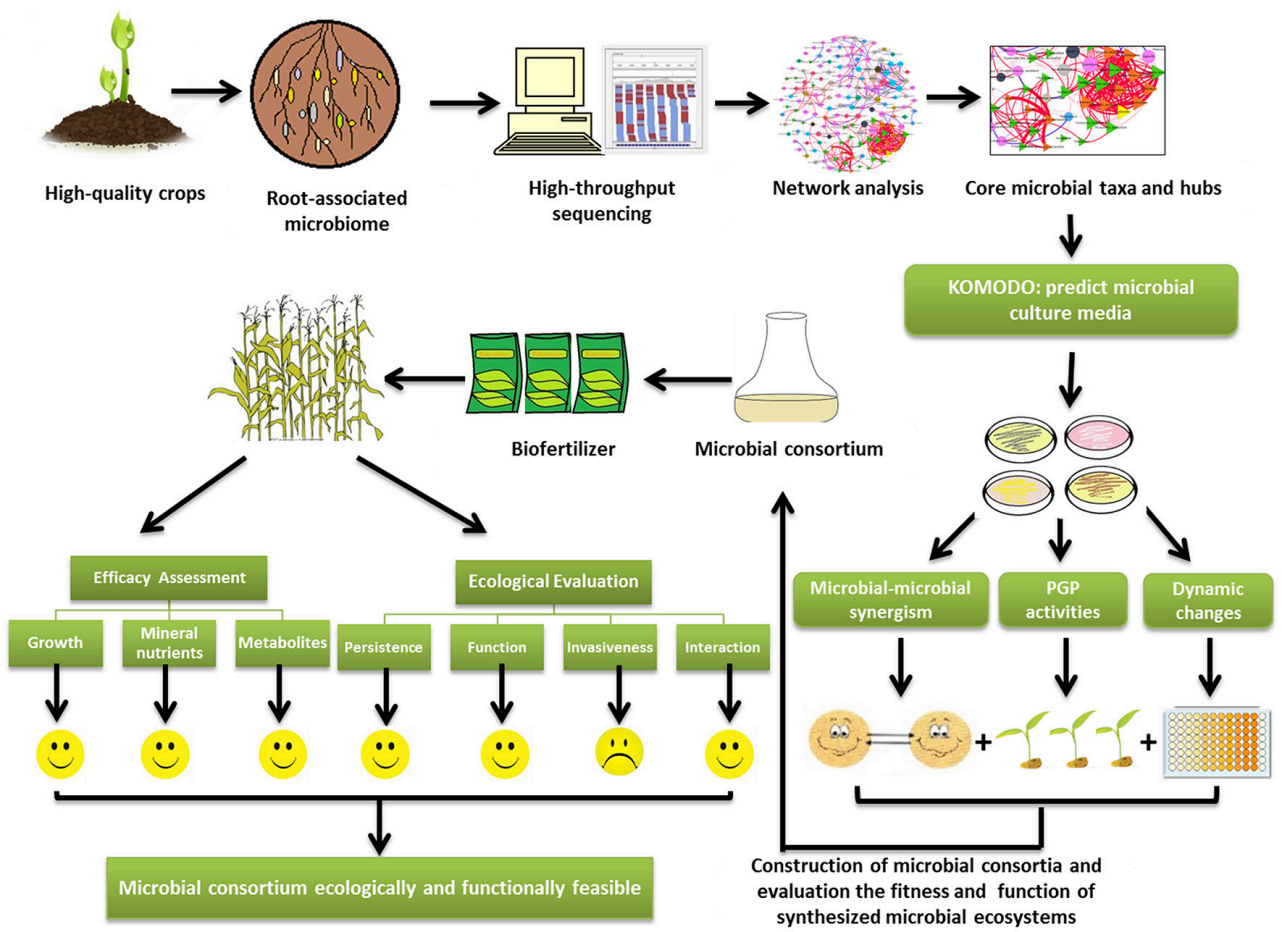
The diagram of technical flow of artificial construction of synthetic microbial consortia (SMC) targeting to augment crop quality. The crops with good quality can be a good origin of SMC. The core microbes can be isolated from the rhizospheric soils or the plant roots of crops with good quality, and their composition can be predicted by next generation sequencing and network analysis. The network analysis will provide the core microbial taxa and hubs which are needed to fulfill ecological services to plants. The web-based platform KOMODO (Known Media Database) can be used to predict the proper medium for the core microbes. The synergism among the microbial members in SMC will be analyzed based on the crop quality (metabolites and nutrients). Plant growth promotion activities and population dynamic changes of the core microbiome are tested to provide reference for constructing the SMC. After the assessment of efficacy and ecological impacts of SMC, they can be utilized in field.

Secondly, how can we obtain the core microorganisms? Now, next generation sequencing (NGS) allows us to perceive the whole microbial community of crops using meta-genomics, which was previously impossible (Figure 1). However, it is unnecessary to inoculate the whole soil microbiome to target fields. The functional redundancy in microbial communities indicates that only the core microbes are needed to fulfill their ecological services to plants (Qin et al., 2016). Microbial network analysis is a powerful tool for identifying the “hubs” (also termed keystone operational taxonomic units), which are highly associated in a microbiome (Banerjee et al., 2018). When basic information about the topology of a microbial network is obtained using the package “igraph” (Csardi and Nepusz, 2006) in R software, the properties of the network structure can be evaluated. Microbial networks can be compartmentalized into several “network modules,” within which microbial species are highly connected with each other. Based on network topological properties, the “hub” species, which coexist with most other species in each module, can be further identified (Toju et al., 2018). These hub species of modules are the candidates of core microorganisms. The information on hub species provides us the very first step in core microorganisms screening.

The next step is to culture these core microbes. As is known, about 99% of the soil microbes cannot be artificially cultured, it is challenging to reproduce all the pure cultures included in SMC. Finding the proper culture media is the key to get the microbial inoculant. Web-based platforms, such as KOMODO (Known Media Database) can be used to predict the media components for culturing the core microbiome (Oberhardt et al., 2015).

Thirdly, the optimization of microbial interactions would be crucial for constructing stable, efficient, and controllable SMC. Cooperation among the SMC members is crucial to exert additive effect in promoting crop quality. Utilizing the positive interactions between fungi (*Trichoderma reesei*) and bacteria, Hu et al. (2017) devised a synergistic SMC with higher lignocellulolytic enzyme activity. The SMC composed of *Enterococcus* and *Clostridium* species degrade wheat straw into hydrogen and butanol in a two-step reaction (Valdez-Vazquez et al., 2015). Constructed SMC using multiple *Escherichia coli* strains successfully assemble 34 proteins in a single culturing, lysis, and purification procedure (Villarreal et al., 2018). The crop quality (metabolites, nutrients) should be integrated as a standard to test the synergistic effect of SMC. The SMC with larger effect than the sum effect of individual microbial taxa, can be regarded as a synergistic SMC. Besides, plant growth promotion activities (ACC deaminase, IAA, siderophores, phosphate solubilization, and etc.), dynamic changes of the core microbes provide reference for constructing a synergistic SMC. Under environmental stresses (such as salinity, drought, or acidity, commonly found worldwide), tolerance traits of the SMC should also be taken into consideration.

## Assessing the efficacy of SMC

The challenges for utilizing SMC in field are the adaptation to new environments. As the SMC are usually isolated from one crop species, they can be expected to positively interact with the same plants in the original soils. A common assumption for field applications is that the microbial inoculants are effective and they have to adapt to a given soil or crop (Rodriguez and Sanders, 2015). Indeed, the variations of soil conditions, such as soil type, moisture, nutrients content, and pH, may affect the functioning and proliferation of SMC. Thus, the efficacy evaluation of SMC mainly focusing on the magnitude in improving crop quality should be conducted in the target field. The strategy from Hart et al. (2018), but also previously used by other authors, as Bona et al. (2016), can be referred to direct the evaluation. The evaluation should start from pot culture in greenhouse involving the factors e.g., soil properties, climatic factors and crop traits (including physiological and phenological traits). Meanwhile, the growth, mineral nutrients and metabolites of crops should be integrated to determine the efficacy of SMC (Figure 1). Following the greenhouse study, plots experiments are subsequently carried out to check the efficacy of the SMC in field. Moreover, the relative longer period (2–3 years) is needed to determine the consistency of the effect of SMC in practice. Based on the above observations, the efficacy of SMC can be obtained.

## Assessing ecological impacts of SMC

In recent years, scientists have developed a much better understanding of how various beneficial soil microbes contribute to plant growth and health. For sustainable development of agricultural ecosystems, it is not only necessary to improve crop yield and quality, but also to ensure a good bioactivity and stability of the soil microbiome in farmland. In this regard, the ecological risks, including invasiveness and the interactions between SMC and indigenous soil microbes should be considered (Hart et al., 2017, 2018). Firstly, the invasiveness should be estimated before releasing SMC in farmland. It should be ascertained how the inoculated strains survive or colonize the rhizosphere of host crops, how the SMC interacts with the indigenous soil microbiome and function, and how the indigenous soil microbiome structurally and functionally responds to the exotic SMC. It is important to clarify all of these issues before implementing SMC on a larger scale in fields. For example, if the introduced soil microbes can inhibit the pathogen populations, the antagonistic interactions would enhance the beneficial effects of SMC to promote the crop health. On the other hand, if the antagonistic interactions occurred between SMC and indigenous beneficial microbes (like AMF and rhizobia), cautions must be taken for using this SMC. Extensive metagenomics and population genomics studies can help assess the environmental impacts of SMC (Rodriguez and Sanders, 2015). With this knowledge in hand, site assessment, potentially ecological risks and regulatory acceptability would all be simplified.

## Concluding remarks

Using SMC is a promising way to improve crop quality in sustainable agriculture. Though abundant studies had shown the positive effects of beneficial soil microbes on the crop yield and quality, the employment of SMC in practices is still infant in developing countries. SMC possesses more merits than individual microbial inoculant. Here, we propose a technical flow of utilizing SMC to promote crop quality. The technical flow starts from how to construct the SMC. The microbes from one crop species with good quality potentially render the same plants higher quality. The core microbes can be isolated from the rhizospheric soils or the plant roots, predicted by next generation sequencing and network analysis. KOMODO can be used to predict the media components for culturing the core microbes. The members of core microbes should be tested for synergy, plant growth promoting activities, and population dynamic changes. The improved crop quality is a main principle for constructing SMC. Further, the efficacy of SMC is needed to test in consideration of the environmental impacts. Finally, the ecological risks evaluation of SMC is essential to maintain the environmental sustainability. The technical flow would be helpful for biostimulant manufacturers and farmers to enhance crop nutritional quality.

## Author contributions

ZK and HL conceived the idea. ZK, HL, and MH prepared the manuscript.

### Conflict of interest statement

The authors declare that the research was conducted in the absence of any commercial or financial relationships that could be construed as a potential conflict of interest. The handling editor declared a past co-authorship with one of the authors ZK.
